# miR-183 inhibits the metastasis of osteosarcoma via downregulation of the expression of Ezrin in F5M2 cells

**DOI:** 10.3892/ijmm.2012.1111

**Published:** 2012-08-24

**Authors:** HAIEN ZHAO, MINGJUN GUO, GUANGYI ZHAO, QIONG MA, BAOAN MA, XIUCHUN QIU, QINGYU FAN

**Affiliations:** Department of Orthopaedic Surgery, Tangdu Hospital, The Fourth Military Medical University, Xi’an, Shaanxi 710038, P.R. China

**Keywords:** osteosarcoma, Ezrin protein, miR-183, migration, invasion

## Abstract

Osteosarcoma is the most common primary malignancy of bone in teenagers and approximately 30% of patients develop lung metastasis, which is the leading cause of mortality. Recent studies suggest that the Ezrin protein is correlated with the metastatic potential of several malignant tumors. In our study, ectopic overexpression of miR-183 repressed the expression levels of Ezrin and significantly inhibited the motility and invasion of osteosarcoma cells. This suggests that miR-183 may possibly play a tumor suppressor role in the metastasis of osteosarcoma by downregulating Ezrin expression levels. These findings show that through inhibition of Ezrin expression levels, miR-183 is significantly involved in cell migration and invasion of osteosarcoma.

## Introduction

Osteosarcoma is the most common primary malignancy of bone in children and adolescents. Clinical data show that this tumor has a poor prognosis, even with the current treatment including amputation and chemotherapy (1,2). During the past 30 years, surgery and neo-adjuvant chemotherapy have been considered as effective treatment approaches for osteosarcoma and have greatly increased limb salvage rate and considerably raised the survival to 65–75%. However, approximately 30% of patients develop lung metastasis, which is the leading cause of mortality (3). Therefore, it is essential to identify metastasis-associated molecules and to better understand the mechanism behind the lung metastasis of osteosarcoma.

microRNAs are a class of small non-coding regulatory RNA molecules, with a profound impact on various biological processes (4–6). It has been reported that microRNAs are aberrantly expressed in most types of cancer where they are considered to play significant roles by regulating the expression of various tumor suppressors and oncogenes (7–9). However, the role of miRNAs in mediating tumor metastasis has only recently been investigated and still remains largely ambiguous.

miR-183 is a member of a miRNA family (miR-183, miR-182 and miR-96) that are clustered within 2–4 kb at chromosome 7q32. miRNAs from this locus are dysregulated in a variety of tumors such as hepatic and colorectal, as well as in leukaemia, lung, and breast cancer (10–13). Furthermore, it has been shown that downregulation of miR-183 is associated with lung cancer metastasis and ectopic overexpression of it inhibits the invasiveness of lung cancer cells (14). Taken together, this suggests that miR-183 plays a significant role in the carcinogenesis or the metastatic cascade, possibly having a tumor suppressor role.

The aim of this study was to investigate the potential role of miR-183 in the invasion and metastasis of osteosarcoma. In this study, we investigated the expression level and functional pattern of miR-183 in osteosarcoma cells. This was performed by quantitation of miR-183 in paired high-metastatic human osteosarcoma F5M2 and low-metastatic human osteosarcoma F4 cells. Functional analysis was then carried out by transfection of miR-183 mimics or inhibitors into the high-metastasis osteosarcoma F5M2 cell line with low endogenous miR-183 expression. The results of the transfection were subsequently assessed on cell viability patterns, cell migration and alterations in gene expression by real-time PCR and in protein levels by western blotting and immunocytochemistry (ICC).

It has been demonstrated that miR-183 regulates the expression of the Ezrin protein, which is involved in controlling actin cytoskeleton, cell adhesion and motility. This is consistent with the cellular function of Ezrin. Taken together, our results suggest that miR-183 plays a significant regulatory role in osteosarcoma cell metastasis, indicating that it might be a novel potential diagnostic and therapeutic target in osteosarcoma.

## Materials and methods

### Cell culture

A pair of human osteosarcoma cell lines with different pulmonary metastatic potentials, high-metastatic F5M2 and low-metastatic F4 cells originating from the human osteosarcoma cell line SOSP-9607 were established in our laboratory (15). The F4 and F5M2 cell lines were maintained in complete RPMI-1640 medium (HyClone) supplemented with 10% fetal calf serum (Sijiqing Co., China) at 37°C with 5% CO_2_.

### Real-time PCR analysis

miR-183, Ezrin mRNA expression was measured by real-time PCR. Total-RNA was extracted by TRIzol reagent (Invitrogen Life Technologies, Carlsbad, CA, USA) according to the manufacturer’s protocol. For miR-183 quantitative real-time PCR, total-RNA was re-transcribed with a miR-specific primer (RiboBio, Guangzhou, China) and then quantitative real-time PCR was performed with a miR-specific primer on the ABI PRISM 7500 real-time PCR system (Applied Biosciences, USA), compared with normalization control U6. Quantitative real-time PCR for Ezrin was performed with primers for Ezrin (forward, 5′-tgggatgctcaaagataatgc-3′ and reverse, 5′-actccaagc caaaggtctgtt-3′) and the relative expression level compared with GAPDH was calculated using the comparative Ct method.

### Transwell insert

We used the Transwell insert (24-well insert; pore size, 8 μm; Corning) to explore the effect of miR-183 on the migration and invasion of F5M2 and F4 cells. Cells suspended in RPMI-1640 medium without fetal bovine serum (FBS) were added to the insert. RPMI-1640 medium with 20% FBS were added to the well out of the insert. After 48 h, the cells on the lower surface of the insert were fixed with 95% ethanol and stained with crystal violet. The invasion assay was performed as the migration assay with the addition of the inserts precoated with 40 μl BD Matrigel (dilution; 1:3; BD Biosciences, San Jose, CA, USA). Then, 6 random visual fields of each insert were counted under a microscope (×40).

### Wound healing assay

Adhered cell monolayers were scratched with a 20 μl pipette tip (Eppendorf) and grown in RPMI-1640 medium with 10% FBS (Sijiqing Co.) at 37°C with 5% CO_2_. Wound healing capacity was monitored by microscopy after 0, 12, 24 and 36 h.

### Apoptosis test

The cells were stained with FITC-conjugated anti-Annexin V antibody. The annexin V-FITC apoptosis detection kit (BD Pharmingen, San Diego, CA, USA) was used to analyze cell apoptosis with flow cytometry (BD Aria; BD Biosciences).

### Western blot analysis and immunocytochemistry

The Ezrin protein was analyzed by western blot analysis using Ezrin Rabbit Monoclonal Antibody and anti-β-actin mouse monoclonal antibody (Epitomics Inc., USA). Immunocytochemistry (ICC) was performed with Ezrin Rabbit Monoclonal Antibody (Epitomics Inc.) and Envision™ Detection kit (Gene Tech, Co., Ltd., Shanghai, China) as standard method.

### Transfection

The transfection was performed with Lipofectamine™ 2000 Reagent (Invitrogen Life Technologies) according to the manufacturer’s instructions. miR-183 mimics, and their negative controls (NCs) and miR-183 inhibitor were purchased from RiboBio. A low concentration of 20 nM or a high concentration of 50 nM of mimics were used for each transfection in the migration, invasion and apoptosis assays, compared with F5M2 cells transfected with NC or with the miR-183 inhibitors. Efficiency of miR-183 transfection was measured by real-time PCR.

### Statistical analysis

All statistical analyses were performed using SPSS 17.0. All data were expressed as the mean ± SD of at least 3 independent experiments. The differences between groups were analyzed using the Student’s t-test; P<0.05 was considered to indicate statistically significant differences.

## Results

### Expression of miR-183 in F5M2 cells is lower than in F4 cells

In this study, the F5M2 and F4 cell lines were selected as the objectives since they originate from the same maternal cell line of human osteosarcoma cell SOSP-9607, but display notable differences in metastatic ability (15).

Of the most potential miRNAs, we focused on miR-183 as it is one of the clearly altered miRNAs and is under-expressed in high-metastatic human pulmonary giant cell carcinoma and colorectal cancer (14,16). However, the functional role of miR-183 in these types of cancer remains unclear.

**Figure 1.**
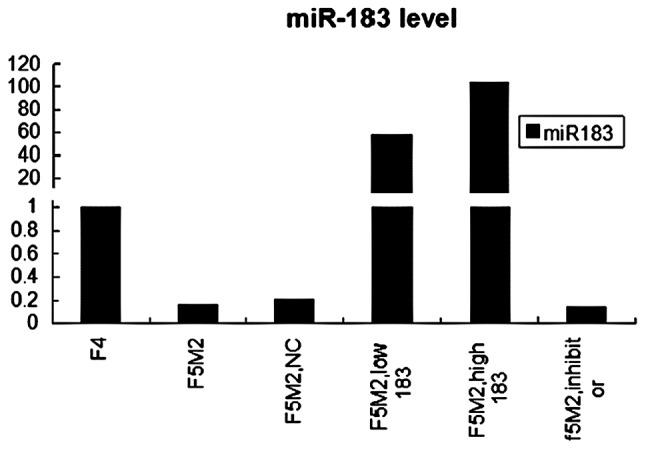
RT-PCR. Total-RNA was isolated from cells and the miR-183 level was analyzed by real-time PCR. miR-183 expression in F5M2 was significantly lower than in F4 cells. miR-183 expression levels increased markedly in F5M2 transfection with miR-183 mimics. However, the levels of miR-183 in F5M2 transfected with NC was not different from that of untreated F5M2 cells. The results represent the relative level of miR-183 in F4.

To study the differential expression of miR-183 in different metastatic potential osteosarcoma cell lines, we employed real-time PCR to compare miR-183 expression between F5M2 and F4. Consistent with the results in pulmonary giant cell carcinoma and colorectal cancer, real-time PCR demonstrated that miR-183 expression in F5M2 was lower than in F4 cells. The difference was statistically significant (P<0.05) (Fig. 1).

### F5M2 significantly overexpresses miR-183 following miR-183 mimic transfection

F5M2 cells were transfected with the miR-183 mimics at a low concentration of 20 nM or a high concentration of 50 nM. Control groups included F5M2 cells that were untreated or transfected with the miR-183 NC or with the miR-183 inhibitors. To examine the efficiency of the transfection, total-RNA was extracted and the miR-183 level was measured by real-time PCR 48 h after transfection.

Real-time PCR showed that miR-183 was significantly over-expressed in F5M2 cells after transfection with the miR-183 mimics, compared with the untreated or treated with mimics NC or the miR-183 inhibitor groups (P<0.05) (Fig. 1). It also showed that miR-183 levels in F5M2 transfection with 50 nM mimics was significantly higher than that of F5M2 20 nM mimics. Real-time PCR demonstrated that the transfection was effective; a higher concentration of miR-183 mimics led to a higher expression of the miR-183.

### miR-183 significantly decreases the migratory and invasive ability of F5M2

In our study, F5M2 cells displayed significantly higher migratory and invasive abilities than F4 cells *in vitro* in a transwell insert experiment, which is in accordance with their metastatic potential (15).

To investigate the possible role of miR-183 in osteosarcoma cell metastasis, we examined the impact on cell motility and invasive ability after ectopic expression of miR-183 in F5M2 cells, which had been verified under expression of endogenous miR-183.

**Figure 2.**
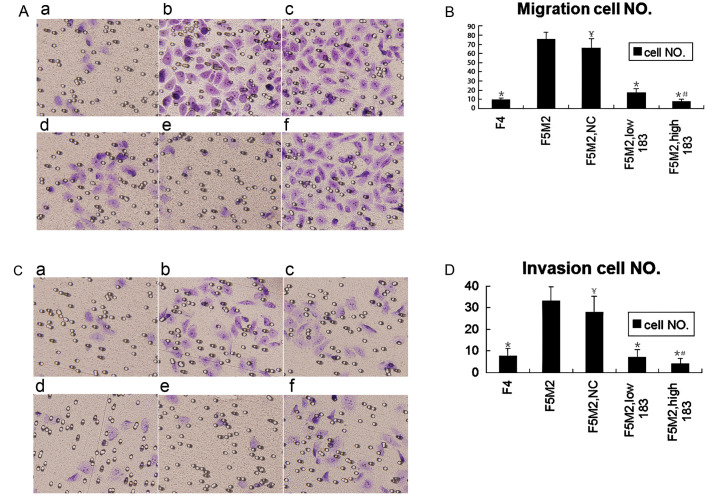
Transwell trials showed that miR-183 regulated F5M2 migration and invasion negatively *in vitro*. (A) Following transfection with or without miR-183 or NC, F5M2 cells suspended in RPMI-1640 medium without fetal bovine serum (FBS) were added to the insert. RPMI 1640 medium with 20% FBS were added to the well out of the insert. After 48 h, the cells on the lower surface of the insert were fixed with 95% ethanol and stained with crystal violet. a, F4; b, F5M2; c, F5M2 with NC; d, F5M2 with low miR-183; e, F5M2 with high miR-183; f, F5M2 with miR-183 inhibitor. (B) The migratory cell number of F4 or F5M2 transfected with miR-183 mimics was lower than that of F5M2 cells untreated or treated with NC. (C) The invasion assay was performed as the migration assay with the addition of the inserts coated with BD Matrigel. a, F4; b, F5M2; c, F5M2 with NC; d, F5M2 with low miR-183; e, F5M2 with high miR-183; f, F5M2 with miR-183 inhibitor. (D) The invasive cell number of F4 or F5M2 transfected with miR-183 mimics was lower than that of F5M2 cells untreated or treated with NC. ^*^P<0.05, compared with F5M2. ^#^P<0.05, compared high miR-183 with low miR-183. ^¥^P>0.05, compared with F5M2.

To examine the migration ability, Transwell insert tests without Matrigel were employed. Cells that penetrated this membrane and reached the underside of the Transwell were counted after 48 h of incubation. The results showed that ectopic expression of miR-183 repressed chemotaxis of F5M2 cells significantly, compared with the NC groups or the untransfected F5M2 cells (Fig. 2A and B) (P<0.05).

To examine the invasion ability, Transwell insert tests with a layer of Matrigel on top of the insert were employed. Cells that penetrated both the Matrigel and membrane were recorded following incubation for 48 h. It showed that miR-183 in F5M2 cells significantly inhibited their invasion (Fig. 2C and D) (P<0.05), which was consistent with the results of migration. Taken together, our results demonstrate that miR-183 inhibited F5M2 cell migration and invasion potential *in vitro*.

Our findings also reveal that the cell motility ability of F5M2 transfection with high concentration mimics was significantly weaker than that of F5M2 with low concentration mimics in both the migration and invasion assay.

### miR-183 significantly decreases the wound healing capacity of F5M2 cells

**Figure 3.**
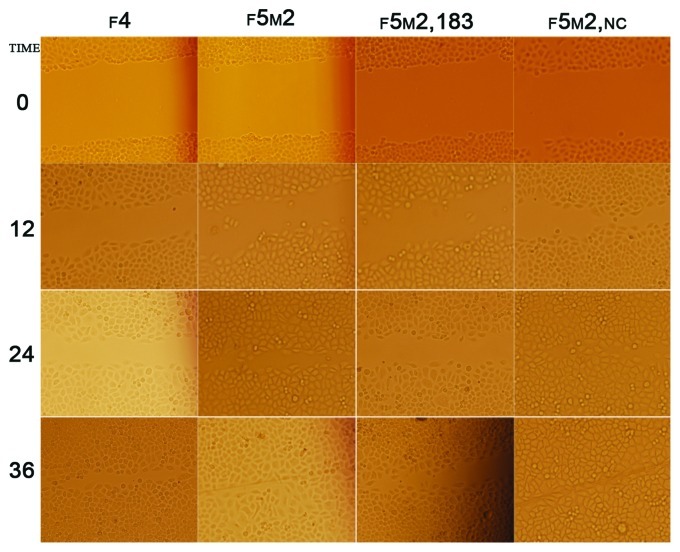
Wound healing assay. Adhered cell monolayers were scratched with a 20 μl pipette tip. Wound healing capacity was monitored by microscopy after 0, 12, 24 and 36 h. The wound closure rate of F4 or F5M2 transfected with miR-183 mimics was significantly lower than that of F5M2 cells untreated or treated with NC.

We employed the scratch wound cell model to compare the polarized migration of F5M2 and F4 cells. The results revealed that F5M2 cells closed the scratch wounds faster than F4 cells (Fig. 3) (P<0.05). This model showed that ectogenic miR-183 significantly decreased the wound healing capacity of F5M2 cells when compared with those cells untreated or transfected with NC (Fig. 3 and Table I). It also showed that inhibition of miR-183 was concentration-dependent.

### miR-183 does not affect apoptosis in F5M2 cells

**Figure 4.**
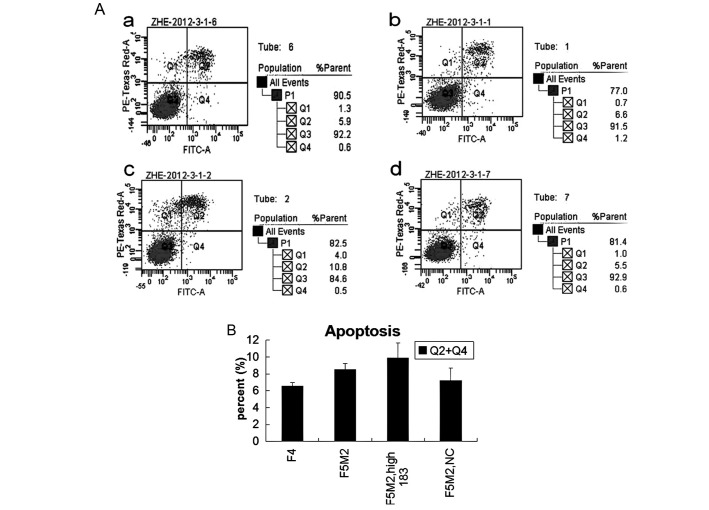
Flow cytometry (FCM) showed that miR-183 has only slight effect on cell apoptosis. (A) Q2 (Quadrant 2) represents the percentage of the viable apoptotic cells. Q4 (Quadrant 4) represents the percentage of the non-viable apoptotic cells. Total apoptotic percentage was equal to Q2 and Q4. a, F4; b, F5M2; c, F5M2 with high miR-183; d, F5M2 with NC. (B) There was no statistically significant difference in total apoptotic ratio between F5M2 and F4 cells, neither between F5M2 cells transfected or un-transfected with miR-183.

The results of apoptosis with flow cytometry showed that there was no statistically significant difference either between F5M2 and F4 cells, or between F5M2 cells transfected with miR-183 mimics and NC or untreated (Fig. 4). Thus, miR-183 has little effect on cell apoptosis and viability.

### miR-183 inhibits the expression of Ezrin

We used 3 miRNA target prediction programs (TargetScan, PicTar and miRanda) to predict the targets of miR-183. Markedly, all 3 programs predicted that VIL2/Ezrin, was the target of miR-183. Therefore, we speculated that miR-183 might alter F5M2 cell migration and invasion by regulating the expression of Ezrin. To verify this speculation, we examined the expression level of Ezrin by western blotting and ICC.

**Figure 5.**
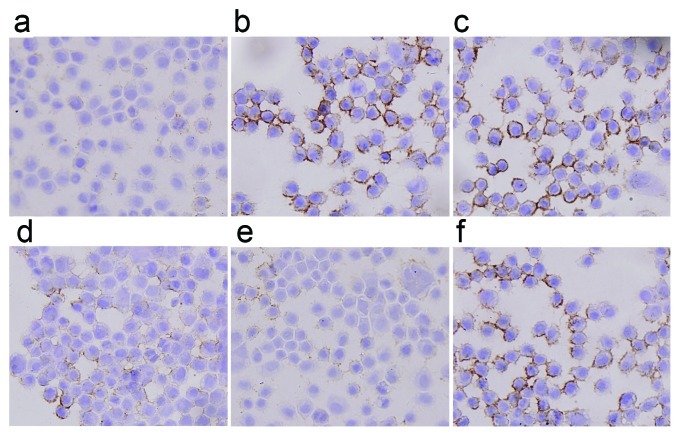
ICC showed that Ezrin expression was significantly higher in F5M2 than in F4 cells. After transfection with miR-183 mimics, Ezrin expression in F5M2 cells was lower than in F5M2 cells untreated or transfected with NC. Ezrin levels in F5M2 cells transfected with a high dose miR-183 was lower than that in F5M2 cells treated with a low dose miR-183. Ezrin was mainly expressed in the membrane of the osteosarcoma. a, F4; b, F5M2; c, F5M2 with NC; d, F5M2 with low miR-183; e, F5M2 with high miR-183; f, F5M2 with miR-183 inhibitor.

**Figure 6.**
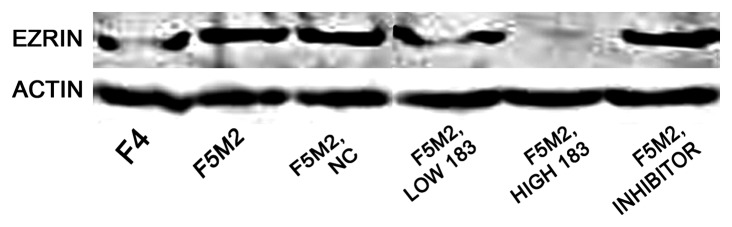
Western blot analysis showed that Ezrin expression was significantly higher in F5M2 than in F4 cells. Following transfection with a low dose miR-183, Ezrin expression was lower than in F5M2 cells untreated or treated with NC. Following transfection with a high dose miR-183, Ezrin expression almost disappeared. It also showed that inhibition of Ezrin expression was dependent on the dose of miR-183. The outcome was consistent with the staining of the ICC. β-actin was used as internal reference positive control.

ICC analysis revealed that staining intensity of Ezrin in F5M2 cells was stronger than in F4 cells and that it decreased greatly after transfection with miR-183 mimics (Fig. 5). Western blotting also showed that the expression level of Ezrin in F5M2 cells was significantly higher than that in F4 cells. Expression of Ezrin in F5M2 cells decreased markedly after transfection with miR-183 mimics, compared with cells untreated or treated with NC (Fig. 6). Both in western blotting and ICC, the expression of Ezrin took on the same tendency; F5M2 cells treated with 50 nM miR-183 mimics expressed less Ezrin than F5M2 cells treated with 20 nM miR-183 mimics (P<0.05). There was an inverse correlation between Ezrin production and miR-183 levels.

## Discussion

miR-183 family members have been shown to be upregulated in colorectal and hepatic tumors, as well as in leukaemia and breast cancer (10–13). By contrast, miR-183 has been shown to be downregulated and inversely correlated with invasive and metastatic ability in pulmonary giant cell cancer (14) and breast cancer (17). Previous studies have demonstrated that the expression profiling of miR-183 was tissue-specific and that it might have divergent functions depending on the tumor tissue or cell type. Previous studies have reported that miRNA repression of mRNA is dependent on the conditions of specific cellular targets (18).

To identify the potential role of miR-183 in osteosarcoma metastasis, we compared miR-183 expression levels in F5M2 and F4 cells, which are high and low metastatic cell lines of osteosarcoma SOSP9607, respectively. We employed multiple approaches to evaluate the inhibitory role of miR-183 in the motility and invasion of F5M2 cells. Consistent with the results in pulmonary giant cell carcinoma and colorectal cancer, real-time PCR demonstrated that miR-183 expression in F5M2 was lower than in F4 cells. Following transfection with miR-183 mimics, the results indicated that overexpression of miR-183 mainly inhibited the migration and invasion of F5M2 cells. Therefore it is possible that miR-183 exerts a suppressing effect on osteosarcoma metastasis, not apoptosis.

It has been reported that several miRNAs, such as miR-335, miR-126, let-7 family, miR-100, miR-218, miR-125, miR-375, miR-142 and miR-198, appear to be metastasis suppressors. Reduced expression of miR-335 and miR-126 were found in breast cancer characterized by poor metastatic-free survival (19), while expression of miR-let7c, miR-100 and miR-218 were significantly decreased in metastatic prostate cancer compared with localized prostate cancer (20). Moreover, ectopic enforced expression of miR-125 impaired cell migration and invasion in a breast cancer cell line and reduction of miR-125 expression enhanced migration of cells (21,22). Ectopic expression of miR-375 induced changes in cell morphology and inhibited melanoma cell invasion and wound healing, strongly suggesting a functional role of miR-375 in cytoskeletal architecture and migration (23). In hepatocellular carcinoma cell lines, the overexpression of miR-142-3p was suppressed, while blocking of miR-142-3p increased migration and invasion. This demonstrates that miR-142-3p expression was downregulated in HCC cells and that miR-142-3p inhibited HCC cell migration and invasion by targeting RAC1 (24). miR-198 was downregulated in hepatocellular carcinoma and forced expression of miR-198 inhibited HCC cell migration and invasion in a c-MET dependent manner (25).

The functional study of miR-183 in malignancy was previously reported in lung and breast cancer cells. Wang *et al* (14) postulated that miR-183 was a potential metastasis inhibitor in lung cancer and reported that upregulation of miR-183 inhibited migration of cancer cells. They demonstrated that miR-183 induced dysregulation of genes related to migration and invasion, including Ezrin. Li *et al* (26) demonstrated that upregulation of miR-183 repressed migration and invasion in HeLa cells, however, this was shown to be mediated via direct targeting of ITGB1. It was indicated that miR-183 was likely to have a number of targets through which it regulated biological functions on cancer cells.

Identification of cancer-specific miRNAs and their targets is pivotal for understanding their role in tumorigenesis and might be important for discovering novel therapeutic targets. To investigate the suppressing mechanism of miR-183 in the metastasis of osteosarcoma, we employed 3 miRNA target prediction programs (TargetScan, PicTar and miRanda) to identify the direct targets of miR-183 (27). Markedly, all the programs predicted that Ezrin was one of the targets of miR-183. Ezrin, which contained the corresponding binding site of miR-183 in Ezrin 3′UTR, could be regulated by miR-183. Several previous studies have demonstrated that miR-183 regulates Ezrin expression in lung cancer cells and Ezrin expression has been associated with tumor invasion and metastasis (14,28). Our study revealed that Ezrin expression was inversely correlated with miR-183, which was consistent with this hypothesis. Our findings also demonstrated that Ezrin levels were positively correlated with osteosarcoma invasion and metastasis. It is reasonable to conclude that alteration of miR-183 might regulate cell migration and invasion targeting the downregulation of Ezrin expression.

Ezrin (also known as cytovillin or villin2) belongs to the ERM family that acts as membrane organizers and linkers between cytoskeleton and plasma membrane (29). Ezrin is a component of cell surface structures that are involved in cell-extracellular matrix interactions as well as in cell-cell interactions (30,31). High expression of the Ezrin protein has been reported to correlate with the metastatic potential of several malignant tumors (32,33).

Ezrin is an interesting molecular marker in osteosarcoma. It has been reported that Ezrin is required for metastasis and recurrence of osteosarcoma (32). Khanna *et al* (34) suggested that Ezrin is a molecule significantly involved in the metastasis of human osteosarcoma and there was a significant association beetween high Ezrin expression and poor outcome in pediatric osteosarcoma. They also reported that a high expression of Ezrin was necessary for metastasis in a mouse osteosarcoma model and high expression of Ezrin was also associated with early pulmonary metastasis in dog osteosarcoma (35,36). Wang *et al* (37) found that expression change of Ezrin was a positive prognostic factor for overall survival and event-free survival in a recent clinical trial. They also concluded that downregulation of Ezrin might be a potential new therapeutic strategy for the treatment of osteosarcoma.

To verify the inhibition of miR-183 on Ezrin expression, we conducted a trial to transfect miR-183 into F5M2 cells, which expressed more Ezrin protein and presented more pulmonary metastatic potential than the paired F4 cells. Notably, following transfection with miR-183 mimics, Ezrin expression in F5M2 cell was almost eradicated either by western blotting or by ICC. By contrast, F5M2 cells following transfection with NC or miR-183 inhibitors changed weakly on Ezrin expression. Our study also showed that ectopic overexpression of miR-183 repressed the motility and invasion of F5M2 cells significantly and acted on the apoptosis or proliferation of F5M2 cells weakly. Taken together, this suggests that miR-183 expression was inversely related to migration and invasion of osteosarcoma cells. Ezrin expression was positively correlated to migration and invasion of osteosarcoma cells. By downregulating the Ezrin expression level, miR-183 might act as a significant inhibitory factor in the progression of osteosarcoma cells.

There have recently been reports regarding miRNAs, identifying miR-20a, miR-222, miR-223, miR-195 and miR-219 as tumor-associated miRNAs, further supporting the hypothesis that miRNAs are involved in tumor metastasis. miR-20a could promote migration and invasion of cervical cancer cells through the direct upregulation of TNKS2, which induced colony formation, migration and invasion of cervical cancer cells (38). Overexpression of miR-223 inhibited proliferation of the cells greatly via the regulation of FOXO1 expression (39). miR-219-5p exerted tumor-suppressive effects in hepatic carcinogenesis through negative regulation of GPC3 expression *in vitro* (40).

To our knowledge, this is the first *in vitro* study to regulate metastasis and progression of osteosarcoma, by upregulation of miR-183 to target the expression of Ezrin in F5M2 cells. The findings of this study illustrate that by downregulating the Ezrin expression level, miR-183 plays a suppressing role in cell migration and invasion of osteosarcoma. Our study might provide an important avenue for further analysis *in vivo* with the aim to develop a new potential diagnostic and therapeutic target for the screening and treatment of high metastatic osteosarcoma. Further studies are required to fully understand the regulation mechanisms of miR-183 and Ezrin in osteosarcoma *in vitro* and *in vivo*.

## Figures and Tables

**Table I.** Wound closure rate of F4 or F5M2 transfected with miR-183 mimics was significantly lower than that of F5M2 cells untreated or treated with NC (P<0.05). There was no significant difference between F5M2 cells untreated or treated with NC (P>0.05). Wound closure rate was defined as closed width/0 h width (means ± SD).

Time	0 h	12 h	24 h	36 h
F4	0	0.44±0.08	0.60±0.02^a^	0.90±0.03^a^
F5M2	0	0.42±0.11	0.82±0.06	0.97±0.03
F5M2, with 183	0	0.41±0.07	0.60±0.08^a^	0.77±0.11^a^
F5M2, NC	0	0.45±0.12^b^	0.85±0.06^b^	0.97±0.03^b^

a P<0.05;

b P>0.05, compared with F5M2 at the same time point;

NC, negative control.
